# Clinical implications of the MELD-XI score in patients undergoing percutaneous coronary intervention: Insights from the SAKURA PCI2 Antithrombotic registry

**DOI:** 10.1016/j.ijcha.2025.101645

**Published:** 2025-03-11

**Authors:** Mitsumasa Sudo, Riku Arai, Keisuke Kojima, Eizo Tachibana, Wataru Atsumi, Michiaki Matsumoto, Naoya Matsumoto, Kazumiki Nomoto, Takaaki Kogo, Ken Arima, Masaru Arai, Yutaka Koyama, Koji Oiwa, Hironori Haruta, Yasuo Okumura

**Affiliations:** a Division of Cardiology, Department of Medicine, Nihon University School of Medicine, Tokyo, Japan; b Department of Cardiology, Kawaguchi Municipal Medical Center, Kawaguchi, Japan; c Department of Cardiology, Nihon University Hospital, Tokyo, Japan; d Division of Cardiology, Department of Medicine, Tokyo Rinkai Hospital, Tokyo, Japan; e Department of Cardiology, Kasukabe Medical Center, Kasukabe, Japan; f Department of Cardiology, Japan Community Health Care Organization Yokohama Chuo Hospital, Yokohama, Japan; g Department of Cardiology, TMG Asaka Medical Center, Asaka, Japan

**Keywords:** Hepatorenal function, Percutaneous coronary intervention, Ischemic heart disease, The Model for End-stage Liver Disease eXcluding International normalized ratio score

## Abstract

**Background:**

The association between hepatorenal function, assessed by the Model for End-stage Liver Disease eXcluding International normalized ratio (MELD-XI) score, and clinical prognosis remains unclear in patients undergoing percutaneous coronary intervention (PCI). The aim of this study was to evaluate the clinical implications of the MELD-XI score.

**Methods:**

Consecutive patients who underwent PCI from June 2020 to September 2022 in the SAKURA PCI2 Antithrombotic registry, a multi-center observational prospective cohort study, were reviewed. Patients with missing data for calculating the MELD-XI score were excluded. Study participating patients were stratified into two groups based on the MELD-XI score: high (>10) and low (≤10). The primary outcome was defined as two-year all-cause mortality. The secondary outcome was defined as major bleeding in accordance with the Bleeding Academic Research Consortium 3 or 5.

**Results:**

Among 1064 patients, 265 (24.9 %) were stratified into a high MELD-XI score. Patients with a high MELD-XI had higher two-year all-cause mortality than those with a low MELD-XI score (19.6 % vs. 4.7 %, log-rank p < 0.01). This association was supported in the multivariable analysis (adjusted HR 3.26, 95 %CI 1.84–5.75, p < 0.01) and further supported by spline curve analysis. A high MELD-XI score was also associated with an increased risk of major bleeding (adjusted HR 2.94, 95 %Ci 1.55–5.56, p < 0.01).

**Conclusions:**

A high MELD-XI score was associated with an increased risk of all-cause mortality and major bleeding within two years. Therefore, the MELD-XI score could provide valuable additional information for risk stratification in patients undergoing PCI.

## Highlights

• The MELD-XI score was related to major bleeding and mortality in PCI patients.
• The c-statistics of the MELD-XI score for bleeding was comparable to other risks.
• The MELD-XI can provide valuable information for risk assessment before the PCI.

## Graphical abstract

**Figure:**
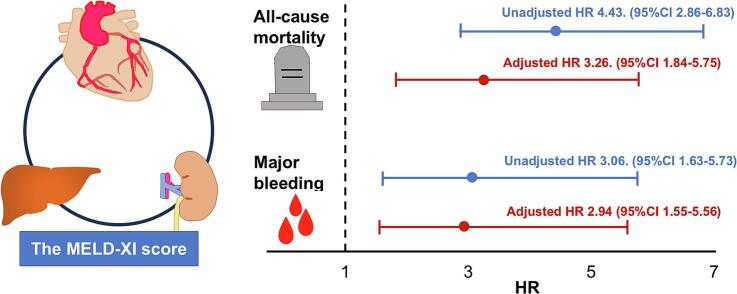

## 1 Introduction

Percutaneous coronary intervention has become an established therapeutic approach for the treatment of ischemic heart disease [1]. Recent advancements in stent technology, imaging modalities, and optimal medical therapy have improved survival rates and alleviated angina symptoms in the contemporary era [[2], [3], [4], [5]]. Nevertheless, various comorbidities continue to influence adverse outcomes such as major bleeding and mortality [[6], [7], [8]]. Among these, renal and liver dysfunction have been identified as crucial factors associated with increased mortality in patients undergoing transcatheter valve replacement or repair, and managing heart failure [[9], [10], [11]].

The Model for End-stage Liver Disease eXcluding International normalized ratio (MELD-XI) score was developed as a marker of liver function in patients with liver cirrhosis and those undergoing liver transplantation [12]. Beyond its initial scope, the MEL-XI score has been recognized as a valuable predictor of prognosis in patients with both hepatic and non-hepatic primary disease [9,10,13]. Nonetheless, the relevance of the MELD-XI score and its clinical implications in patients undergoing percutaneous coronary intervention (PCI) remains unclear. Therefore, this study aimed to evaluate the association of the MELD-XI score with clinical outcomes in patients undergoing PCI.

## 2 Methods

### 2.1 Study population

The present study was conducted as a retrospective analysis of data from the SAKURA PCI2 Antithrombotic Registry, a multi-center observational prospective cohort study [14]. Data of patients who underwent PCI at seven medical centers in Japan − Nihon University Itabashi Hospital, Nihon University Hospital, Kawaguchi Municipal Medical Center, Tokyo Rinkai Hospital, Kasukabe Medical Center, Japan Community Health care Organization Yokohama Chuo Hospital, and TMG Asaka Medical Center − between June 2020 and September 2022 were reviewed. Patients with missing data required to calculate the MELD-XI score were excluded from the present study. This study was conducted according to the Declaration of Helsinki and was approved by the institutional review boards of all participating centers (RK200310-3). This trial was registered with the University Hospital Medical Information Network (UMIN Clinical Trials Registry: UMIN000041849). All patients provided written informed consent to the procedure and data collection.

### 2.2 Assessment of the hepatorenal function

Blood examinations were routinely performed before the PCI procedure. The MELD-XI score was calculated based on the following formula: 5.11 × ln (serum bilirubin [mg/dL]) + 11.76 × ln (serum creatinine [mg/dL]) + 9.44 [12]. A high MELD-XI score was defined as > 10, based on a previous study [9].

### 2.3 Assessment of the high bleeding risk

The Academic Research Consortium for High Bleeding Risk (ARC-HBR) and the CREDO-Kyoto Bleeding Risk Score were proposed as a marker to assess bleeding risk in patients undergoing PCI [15,16]. The ARC-HBR consists of major and minor criteria. Patients who met one major or two minor criteria were considered high bleeding risk patients. The ARC-HBR total score was calculated as 1 point for major criteria and 0.5 points for each minor criterion. The CREDO-Kyoto Bleeding Risk Score consists of seven variables. Patients scoring three or more points were classified as high bleeding risk.

### 2.4 Clinical outcomes

All clinical events were followed up through a central registry office and obtained by medical records or reports on follow-up questionnaires sent to patients. The primary outcome was defined as all-cause mortality within two years. The secondary outcome was major bleeding, defined as the Bleeding Academic Research Consortium type 3 or 5 [17]. In brief, type 3a was defined as any transfusion with overt bleeding or overt bleeding plus hemoglobin drop ≥ 3 to <5 g/dL (provided hemoglobin drop is related to bleeding). Type 3b included overt bleeding plus hemoglobin drop ≥ 5 g/dL (provided hemoglobin drop is related to bleed), cardiac tamponade, bleeding requiring surgical intervention for control (excluding dental/nasal/skin/hemorrhoid), and bleeding requiring intravenous vasoactive drugs. Type 3c referred to intracranial hemorrhage (does not include microbleeds or hemorrhagic transformation; does include intraspinal) and intraocular bleed compromising vision. Type 5 was defined as fatal bleeding.

### 2.5 Statistical analysis

Categorical variables are presented as numbers with percentages. Continuous variables are presented as means with standard deviations or medians with interquartile ranges (IQR). Inter-group differences in categorical variables were analyzed using the Chi-square test or Fisher’s exact test. Inter-group differences in continuous variables were analyzed using the unpaired Student’s *t*-test or the Wilcoxon rank-sum test.

The Kaplan–Meier method was used to estimate the rates of clinical outcomes. A log-rank test was applied to compare outcomes between the groups. Univariate and multivariable Cox proportional hazards regression analyses were conducted to calculate hazard ratios (HRs) and 95 % confidence intervals (95 % CIs) for the clinical outcomes.

The association between the primary outcome and the MELD-XI score was adjusted in the multivariable model, including predefined covariates based on previous clinical knowledge, such as sex, age, diabetes mellitus, peripheral artery disease, chronic kidney disease, left ventricular ejection fraction, statin use, and clinical presentation [[18], [19], [20], [21]]. Using a spline curve, the association between the MELD-XI score and the primary outcome was illustrated. Additionally, a time-dependent receiver operating characteristic (ROC) analysis was performed, and c-statistics were used to assess the optimal predictive value for the primary outcome.

Furthermore, potential interactions between the MELD-XI score and the following subgroups on the primary outcome were examined: sex (male vs. female), age (≥75 years vs. <75 years), diabetes mellitus (no vs. yes), renal function (estimated glomerular filtration rate ≥ 60 mL/min/1.73 m^2^ vs. < 60 mL/min/1.73 m^2^), left ventricular function (≥40 % vs. <40 %), peripheral artery disease (no vs. yes), statin (no vs. yes), clinical presentation (chronic coronary syndrome vs. acute coronary syndrome) and clinical frailty scale (≥5 vs. < 5).

The association between the secondary outcome and the MELD-XI score was adjusted for sex and age. A spline curve also illustrated the association between the MELD-XI score and the secondary outcome. Potential interactions between the MELD-XI score and the following subgroups about the secondary outcome were examined: sex (male vs. female), age (≥75 years vs. < 75 years), access site for PCI (radial artery vs. non-radial artery), and planned dual antiplatelet therapy (DAPT) duration (≤3 months vs. > 3 months). The Spearman rank correlation coefficient was used to assess a correlation between the MELD-XI score and the ARC-HBR total score. A time-dependent ROC analysis was performed, and c-statistics were used to evaluate the optimal predictive value of the MELD-XI score and the ARC-HBR for the secondary outcome.

To examine the robustness of our inference, we performed a sensitivity analysis. Patients were divided into three groups based on the tertile of the MELD-XI score. The Kaplan-Meier analysis was conducted to compare the third tertile with the first and second tertiles. Finally, we conducted a propensity score matching analysis. Patients in our cohort were matched between two groups using propensity scores. The variables used to calculate the propensity score included all variables from the comparative analysis, except for liver and renal function and hemodialysis. After matching, Kaplan-Meier analysis and Cox proportional hazards regression were conducted to assess the association between the MELD-XI score and the primary outcome. A two-tailed p-value of < 0.05 was accepted as statistically significant. All statistical analyses were performed using JMP pro 17 (version 17.2.0; SAS Institute Inc, Cary, NC, USA) and R (version 4.1.1; R Foundation for Statistical Computing, Vienna, Austria).

## 3 Results

### 3.1 Study population

A total of 1064 patients were analyzed in the present study. Overall, 78.5 % of the patients were male, and the mean age was 69.9 ± 11.7 years old. The median MELD-XI score was 6.1 (IQR 3.2, 10.1) (Table 1). Based on the MELD-XI score, a high MELD-XI score was identified in 265 patients (24.9 %), while the remaining 799 patients (75.1 %) had a low MELD-XI score. Patients with a high MELD-XI score were older (71.8 ± 11.1 vs. 69.2 ± 11.9 years, p < 0.01), more likely to be male (84.5 % vs. 76.5 %, p = 0.01), and had more frequently diabetes mellitus (61.5 % vs. 41.6 %, p < 0.01), peripheral artery disease (17.4 % vs. 4.8 %, p < 0.01), hemodialysis (39.6 % vs. 0.5 %, p < 0.01), and non-radial artery access (62.1 % vs. 28.1 %, p < 0.01) (Table 1).

**Table 1:** Patient characteristics.

	Overall (n = 1064)	High MELD-XI (n = 265)	Low MELD-XI (n = 799)	p-value
Male, n (%)	835 (78.5)	224 (84.5)	611 (76.5)	<0.01
Age, years	69.9 ± 11.7	71.8 ± 11.1	69.2 ± 11.9	<0.01
Hypertension, n (%)	792 (74.6)	224 (84.5)	570 (71.3)	<0.01
Diabetes Mellitus, n (%)	495 (46.5)	163 (61.5)	332 (41.6)	<0.01
Dyslipidemia, n (%)	658 (61.8)	155 (58.5)	503 (63.0)	0.19
Prior myocardial infarction, n (%)	179 (16.9)	61 (23.0)	118 (14.8)	<0.01
Prior PCI, n (%)	293 (27.5)	111 (41.9)	182 (22.8)	<0.01
Prior CABG, n (%)	35 (3.3)	12 (4.5)	23 (2.9)	0.19
Atrial fibrillation, n (%)	129 (12.2)	41 (15.6)	88 (11.1)	0.06
Peripheral artery disease, n (%)	84 (7.9)	46 (17.4)	38 (4.8)	<0.01
Clinical presentation				0.16
Chronic coronary syndrome, n (%)	490 (46.1)	132 (49.8)	358 (44.8)	
Acute coronary syndrome, n (%)	574 (54.0)	133 (50.2)	441 (55.2)	
Clinical frailty scale ≥ 5, n (%)	73 (6.9)	31 (11.7)	42 (5.3)	<0.01
Oral anticoagulants or DOAC, n (%)	137 (12.9)	42 (15.9)	95 (11.9)	0.10
Statin, n (%)	921 (86.6)	214 (80.8)	707 (88.5)	<0.01
Hemodialysis, n (%)	109 (10.2)	105 (39.6)	4 (0.5)	<0.01
Creatinine, mg/dL	0.9 (0.8, 1.3)	2.7 (1.4, 7.3)	0.8 (0.7, 1.0)	<0.01
eGFR, ml/min/1.73 m^2^	59.7 (41.2, 75.6)	19.4 (6.3, 38.9)	66.8 (55.3, 80.0)	<0.01
Total Bilirubin, mg/dL	0.6 (0.4, 0.8)	0.5 (0.3, 1.0)	0.6 (0.4, 0.8)	0.04
Left ventricular ejection fraction, %	59 (46, 66)	53 (40, 62)	60 (49, 67)	<0.01
Access site for PCI				<0.01
Radial artery, n (%)	672 (63.5)	99 (37.8)	573 (71.9)	
Non-radial artery, n (%)	387 (36.5)	163 (62.1)	224 (28.1)	
Planned DAPT duration ≤ 3 months, n (%)	598 (56.3)	133 (50.2)	465 (58.3)	0.02

Categorical variables are presented as absolute numbers and percentages. Continuous variables are presented as the mean ± standard deviation or as the median and interquartile range.

CABG, Coronary artery bypass graft; eGFR, estimated glomerular filtration rate; DAPT, Dual antiplatelet therapy; DOAC, Direct oral anticoagulants; PCI, Percutaneous coronary intervention.

### 3.2 The association between the MELD-XI score and clinical outcomes

**Fig. 1:**
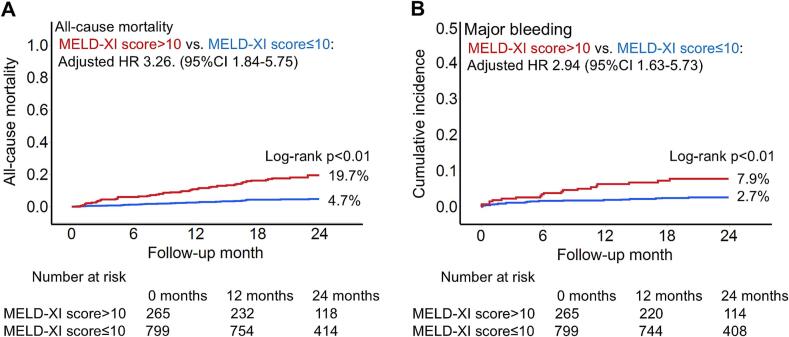
The Kaplan–Meier curves for all-cause mortality and major bleeding within two years. The Kaplan–Meier curves showed that patients with a high MELD-XI score had higher estimated all-cause mortality (19.7 % vs. 4.7 %, log-rank p < 0.01) (A) and major bleeding (7.9 % vs. 2.7 %, log-rank p < 0.01) (B) within two years compared to those with a low MELD-XI score. MELD-XI score, Model for End-stage Liver Disease eXcluding International normalized ratio score.

**Fig. 2:**
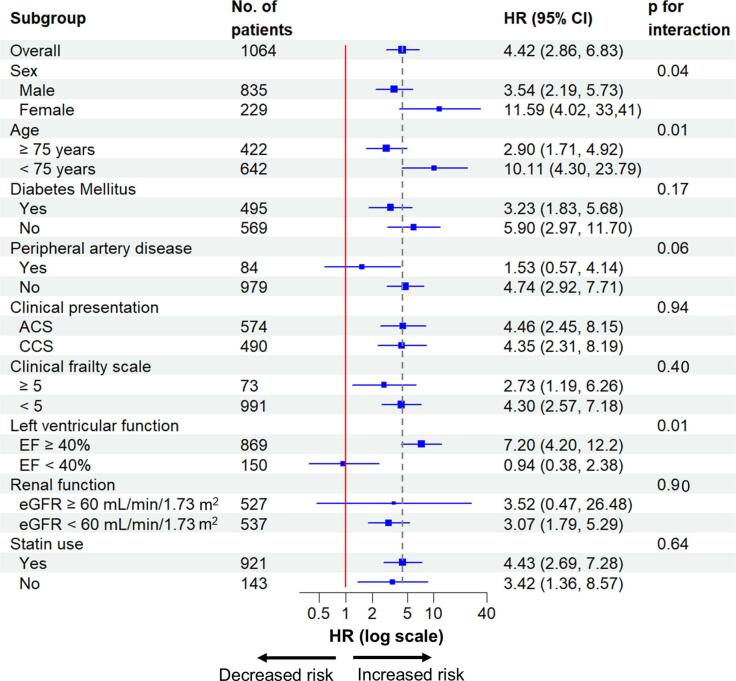
Subgroup analysis of the primary outcome in patients with a high MELD-XI score. A forest plot illustrates hazard ratios for two-year all-cause mortality after PCI in patients with a high MELD-XI score. In each subgroup, hazard ratio and 95% confidence intervals are presented. ACS, acute coronary syndrome; CCS, chronic coronary syndrome; CI, confidence interval; EF, ejection fraction; eGFR, estimated glomerular filtration rate; HR, hazard ratio; MELD-XI score, Model for End-stage Liver Disease eXcluding International normalized ratio score.; PCI, percutaneous coronary intervention.

The median follow-up duration was 24.3 months (IQR 18.5, 31.9). During the two-year follow-up, 83 patients died. Of these, 19 (22.9 %) were attributed to cardiovascular causes, three (3.6 %) to cerebrovascular causes, and 61 (73.5 %) to other causes (Sup. Table). All-cause mortality within two years was higher in patients with a high MELD-XI score compared to those with a low MELD-XI score (19.7 % vs. 4.7 %, log-rank p < 0.01) (Fig. 1A). In the univariate Cox regression hazard model, a high MELD-XI score was associated with an increased risk of all-cause mortality (HR 4.42, 95 %CI 2.86–6.83, p < 0.01), which remained consistent in the multivariable model (HR 3.26, 95 %CI 1.84–5.75, p < 0.01). Additionally, the MELD-XI score as a continuous variable was associated with all-cause mortality (unadjusted HR 1.06, 95 %CI 1.04–1.09, p < 0.01, adjusted HR 1.06, 95 %CI 1.03–1.09, p < 0.01), a finding further supported by a spline curve (Table 2, Sup. Fig. S1A). Furthermore, a time-dependent ROC analysis showed that the c-statistics for predicting primary outcome were 0.69 (95 %CI 0.62–0.76). The association between the MELD-XI score and all-cause mortality was consistent across clinical subgroups, including clinical presentation, statin use, and clinical frailty scale, with the exception of left ventricular ejection fraction (Fig. 2).

**Table 2:** Association of a high MELD-XI score with primary and secondary outcome.

	Univariate analysis				Multivariable analysis		
	HR	95 %CI	p-value		HR	95 %CI	p-value
Primary outcome							
High MELD-XI score	4.43	2.86–6.83	<0.01		3.26	1.84–5.75	<0.01
Age (per 1 year increase)	1.07	1.04–1.09	<0.01		1.07	1.04–1.09	<0.01
Male	1.16	0.67–2.00	0.59		1.19	0.68–2.08	0.55
Diabetes mellitus	1.76	1.14–2.74	0.01		1.37	0.87–2.18	0.17
Peripheral artery disease	3.13	1.84–5.33	<0.01		1.48	0.83–2.64	0.19
eGFR < 60 mL/min/1.73 m^2^	3.65	2.17–6.15	<0.01		1.04	0.53–2.04	0.91
LVEF < 40 %	1.98	1.19–3.27	<0.01		1.52	0.91–2.53	0.11
Statin use	0.45	0.27–0.75	<0.01		0.60	0.36–1.03	0.06
Acute coronary syndrome	0.94	0.61–1.44	0.77		1.11	0.71–1.74	0.64
Secondary outcome							
High MELD-XI score	3.06	1.63–5.73	<0.01		2.94	1.55–5.56	<0.01
Age (per 1 year increase)	1.04	1.01–1.08	0.01		1.04	1.00–1.07	0.02
Male	0.80	0.39–1.64	0.54		0.82	0.39–1.72	0.60

CI, confidence interval; eGFR, estimated glomerular filtration rate; LVEF, Left ventricular function; HR hazard ratio; MELD-XI score, Model for End-stage Liver Disease eXcluding International normalized ratio score.

Regarding the secondary outcome, patients with a high MELD-XI score had a higher rate of major bleeding within two years than those with a low MELD-XI score (7.9 % vs. 2.7 %, p < 0.01) (Fig. 1B). In univariate analysis, a high MELD-XI score was associated with an increased risk of major bleeding (HR 3.06, 95 %CI 1.63–5.73, p < 0.01), which was consistent after adjustment for sex and age (HR 2.94, 95 %CI 1.55–5.56, p < 0.01) (Table 2). Similarly, the MELD-XI score as a continuous variable was associated with major bleeding (adjusted HR 1.06, 95 %CI 1.03–1.10, p < 0.01), a finding further supported by a spline curve (Sup. Fig. S1B). The association between the MELD-XI score and major bleeding was consistent across clinical subgroups, including PCI access site and planned DAPT duration (Sup. Fig. S2).

### 3.3 The association between the MELD-XI score and the ARC-HBR

The MELD-XI score was positively correlated with the ARC-HBR total score (R = 0.49, p < 0.01) and the CREDO-Kyoto Bleeding Risk Score (R = 0.55, p < 0.01). According to the time-dependent ROC analysis, the c-statistics of the MELD-XI score for predicting major bleeding within two years were similar to those of the ARC-HBR total score (0.67 vs. 0.74, p = 0.10) and the CREDO-Kyoto Bleeding Risk Score (0.67 vs. 0.69, p = 0.74).

### 3.4 Sensitivity analysis

Patients were stratified into three groups based on tertiles of the MELD-XI score: the first tertile (MELD-XI ≤ 4), the second tertile (4 < MELD-XI ≤ 8), and the third tertile (8 < MELD-XI). Kaplan-Meier curves showed that the two-year all-cause mortality was higher in the third tertile than the first and second tertiles (first vs. second vs. third; 4.0 % vs. 4.6 % vs. 15.6 %, log-rank p < 0.01). In the propensity score matching analysis, a high MELD score remained significantly associated with two-year all-cause mortality (18.0 % vs. 6.1 %, log-rank p < 0.01; HR 2.95, 95 % CI 1.53–5.71, p < 0.01).

## 4 Discussions

The present study clarified the clinical implications of hepatorenal function, as assessed by the MELD-XI score, in patients undergoing percutaneous coronary intervention. The findings of this observational study are summarized as follows:

1. The MELD-XI score was independently associated with all-cause mortality within two years after PCI.
2. The MELD-XI score as a continuous variable was correlated with the ARC-HBR total score and the CREDO-Kyoto Bleeding Risk Score, and a high MELD-XI score was associated with an increased risk of major bleeding within two years.

In the contemporary era, PCI has been established as a standard treatment for ischemic heart disease, particularly for improving mortality in patients with acute coronary syndrome [1]. Moreover, in symptomatic patients with stable angina, PCI has been shown to effectively reduce angina symptoms [2]. Nevertheless, many patients who received PCI have comorbidities that contribute to the worsening clinical course. Therefore, Therefore, effective risk stratification before invasive treatment is essential to facilitate clinical management. In particular, assessing the risk of major bleeding is crucial in considering the optimal duration of DAPT after PCI. To our knowledge, this study is the first to report that the MELD-XI score, reflecting hepatorenal function, can predict both all-cause mortality and major bleeding.

The MELD-XI score was originally developed as a risk assessment tool for transplantation in patients with end-stage liver disease [12]. With serum bilirubin and serum creatinine in its formula, this score has recently been thought to reflect hepatorenal function. In patients with heart failure or valvular disease, the MELD-XI score was associated with right-sided heart volume overload and increased mortality [22]. Similarly, a high MELD-XI score in patients with acute coronary syndrome was associated with heart failure re-hospitalization and mortality in small population studies [23,24]. This finding aligns with our results, shown in the subgroup analysis. Furthermore, the MELD-XI score was also found to be relevant to mortality in patients with chronic coronary syndrome in the present study. According to a time-dependent ROC analysis, the MELD-XI score exhibited acceptable predictive performance for all-cause mortality. The possible explanation of the mechanism is that renal and liver dysfunctions cause atherosclerosis progression. Specifically, liver dysfunction leads to increased oxidative stress and reduced nitric oxide, resulting in endothelial dysfunction, increased tissue factor production, vascular smooth-nuclear proliferation, and increased systemic inflammation driven by the overexpression of an inflammatory cytokine such as TNF-α. Consequently, liver dysfunction accelerates atherosclerosis progression [25]. Similarly, renal dysfunction increases plasma levels of pro-inflammatory cytokines, including interleukin-6, tumor necrosis factor-α, and monocyte chemotactic protein-1, promoting vascular inflammation. Additionally, reduced nitric oxide synthesis contributes to endothelial dysfunction., further driving atherosclerosis progression [26]. Given these mechanisms, it is reasonable to consider that hepatorenal dysfunction contributes to atherosclerotic changes in peripheral arteries and the cerebrocardiovascular system, ultimately leading to fatal atherosclerotic diseases [23,27,28]. In addition, the potential for right-sided heart volume overload in patients with a high MELD-XI linked to heart failure exacerbation may contribute to increased mortality risk [22]. In the present study, patients with a high MELD-XI score also experienced major bleeding related to poor prognosis [29]. Considering the above, it is unsurprising that the MELD-XI as a marker reflecting renal and hepatic function is independently associated with clinical outcomes.

In the current study, patient characteristics differed between two groups. Patients with a high MELD-XI score had more comorbidity linked to worse outcomes, such as diabetes mellitus, frailty, and low left ventricular ejection fraction, compared to those with a low MELD-XI score [20,21,23]. Additionally, patients with a MELD-XI score had lower rates of statin use associated with a favorable prognosis in patients with ischemic heart disease. Therefore, several analyses were conducted to ensure our findings' robustness. The multivariable analysis confirmed that the MELD-XI score as a continuous variable was associated with all-cause mortality. As reliance on a single cut-off value may not fully capture the nonlinear associations between MELD-XI and outcomes, we conducted both spline and tertile-based analyses as a sensitivity analysis in the present study. These analyses supported that a high MELD-XI score was associated with an increased mortality risk. Moreover, a high MELD-XI score consistently showed an increased mortality risk across subgroups, except for a low left ventricular ejection fraction. Notably, a high MELD-XI score consistently was related to all-cause mortality, even in patients with preserved renal function and those receiving statin therapy. This finding suggests that patients with a high MELD-XI score require closer follow-up after PCI. In multivariable analysis, statin use was independently associated with a favorable outcome in the present study, highlighting the potential benefit of statin therapy in improving the prognosis for patients with a high MELD-XI score (Table 2).

Regarding the secondary outcome, the MELD-XI score was associated with major bleeding in the present study. This association consistently remained after adjustment and was further supported by spline curves. The possible underlying mechanism is that chronic renal and liver dysfunctions are linked to reduced platelet function and thrombocytopenia. Specifically, in renal dysfunction, disturbance of the platelet α-granules causes insufficient platelet function. Additionally, oxidative stress and inflammation in renal dysfunction impair platelet adhesion and aggregation [30]. Similarly, liver dysfunction contributes to platelet dysfunction through multiple mechanisms, including splenomegaly, splenic sequestration, relatively reduced thrombopoietin, consumption owing to autoantibodies, and bone marrow suppression [31]. Consequently, renal and liver dysfunctions increase the risk of major bleeding, such as gastrointestinal bleeding [32,33]. Thus, it seems reasonable that our finding was associated with major bleeding.

In the present study, patients with a high MELD-XI were more likely to be accessed using a non-radial artery and planned DAPT duration > 3 months. Both non-radial artery access and prolonged DAPT duration are associated with an increased risk of bleeding events. They may contribute to the occurrence of major bleeding in patients with a high MELD-XI score [34]. Although a high MELD-XI score was associated with major bleeding regardless of PCI access site and planned DAPT duration in a subgroup analysis, non-radial artery access and prolonged DAPT duration appeared to pose a higher risk. Therefore, to mitigate the risk of major bleeding risk, radial artery access and a shorter planned DAPT duration should be considered in patients with a high MELD-XI.

The MELD-XI was correlated with the ARC-HBR total score and the CREDO-Kyoto Bleeding Risk Score, and the c-statistics for predicting major bleeding were comparable between the MELD-XI score and these indicators. The ARC-HBR and the CREDO-Kyoto Bleeding Risk Score require checking multiple criteria, which can be challenging in clinical settings [15]. Thus, the MELD-XI score, calculated using only serum creatine and total bilirubin levels, can provide a more accessible assessment tool for predicting major bleeding before the PCI procedure.

It should be noted that the limitation of the MELD-XI score is that it was originally developed to evaluate liver function in end-stage liver disease. Therefore, its applicability in assessing early-stage liver fibrosis remains uncertain. However, the MELD-XI score has the advantage of simplicity, allowing for an easily combined assessment of liver and renal function, and has the potential for broader clinical use. Most importantly, in the present study, it was found to be significantly associated with both mortality and bleeding in patients undergoing PCI. Accordingly, the MELD-XI could provide valuable additional information for identifying vulnerable patients at risk of unfavorable outcomes after the PCI procedures. Furthermore, it may aid in the decision-making regarding the optimal duration of DAPT.

## 5 Limitations

Several limitations should be acknowledged in the present study. First, this study was conducted as a retrospective analysis of data from a multicenter prospective observational cohort study in Asia, with 78.5 % of the participants being male. Therefore, our findings might be subject to selection bias, and their generalizability should be evaluated in more diverse populations. Second, since the MELD-XI score was not reassessed after the PCI procedure, further studies are needed to clarify whether improvement in the MELD-XI score impacts clinical outcomes. Third, follow-up duration and sample size were limited. As a result, late outcomes, such as recurrent cardiovascular events and chronic bleeding complications, were not collected. Fourth, inflammatory biomarkers such as C-reactive protein and interleukin-6 were not collected, nor was thrombopoietin a marker of hepatic synthetic function. Thus, the present study was limited in exploring the mechanism of atherosclerosis and bleeding diathesis in patients with hepatorenal dysfunction. To validate our findings, large-scale prospective observational studies with long-term follow-up, including non-Asian populations and more heterogeneous cohorts, are required. Fifth, given the simplicity of the MELD-XI formula, it may not fully capture the complexity of hepatorenal dysfunction and could underestimate its severity in certain cases. Nevertheless, this study provided valuable insights into the association of the MELD-XI score with all-cause mortality and major bleeding in patients undergoing PCI.

## 6 Conclusion

A high MELD-XI score exhibited an increased risk of all-cause mortality and major bleeding within two years. Assessing hepatorenal function may offer valuable additional information for risk stratification and clinical management in patients undergoing PCI.

## Declaration of generative AI and AI-assisted technologies in the writing process

During the preparation of this work the authors used OpenAI’s ChatGPT in order to enhance English expressions. After using this tool, the authors reviewed and edited the content as needed and take full responsibility for the content of the published article.

## CRediT authorship contribution statement

**Mitsumasa Sudo:** Writing – original draft, Visualization, Resources, Methodology, Investigation, Formal analysis, Conceptualization. **Riku Arai:** Writing – review & editing, Project administration, Investigation, Data curation. **Keisuke Kojima:** Writing – review & editing, Resources. **Eizo Tachibana:** Writing – review & editing, Resources. **Wataru Atsumi:** Writing – review & editing, Resources, Investigation, Data curation. **Michiaki Matsumoto:** Writing – review & editing, Resources, Investigation, Data curation. **Naoya Matsumoto:** Writing – review & editing, Resources. **Kazumiki Nomoto:** Writing – review & editing, Resources. **Takaaki Kogo:** Writing – review & editing, Resources, Investigation, Data curation. **Ken Arima:** Writing – review & editing, Resources. **Masaru Arai:** Writing – review & editing, Investigation, Data curation. **Yutaka Koyama:** Writing – review & editing, Resources, Investigation, Data curation. **Koji Oiwa:** Writing – review & editing, Resources. **Hironori Haruta:** Writing – review & editing, Resources, Investigation, Data curation. **Yasuo Okumura:** Writing – review & editing, Supervision.

## Declaration of competing interest

The authors declare the following financial interests/personal relationships which may be considered as potential competing interests: [Yasuo Okumura reports a relationship with Johnson & Johnson KK, Biosense Webster, Inc., Nippon Boehringer Ingelheim, remuneration from Daiichi-Sankyo, AstraZeneca, Bayer Healthcare, Bristol-Myers Squibb that includes: funding grants. Yasuo Okumura belongs to the endowed departments of Boston Scientific Japan, Biotronik Japan, Abbott Medical Japan, Japan Lifeline, and Medtronic Japan. If there are other authors, they declare that they have no known competing financial interests or personal relationships that could have appeared to influence the work reported in this paper.]
