# Supplementary material for: Clinical implications of the MELD-XI score in patients undergoing percutaneous coronary intervention: Insights from the SAKURA PCI2 Antithrombotic registry
Source: Int J Cardiol Heart Vasc. 2025 Mar 11;57:101645. doi: 10.1016/j.ijcha.2025.101645 (PMC11932686; doi:10.1016/j.ijcha.2025.101645)
Supplement: Supplementary Data 1 [file mmc1.docx]

**Supplemental Table. Details of two-year all-cause mortality**

|  | n = 83 |
| --- | --- |
| Cardiovascular causes | 19 (22.9%) |
| Cerebrovascular causes | 3 (3.6%) |
| Other causes | 61 (73.5%) |
| Renal failure | 5 (6.0%) |
| Sepsis | 2 (2.4%) |
| Pneumonia | 1 (1.2%) |
| Alveolar hemorrhage | 1 (1.2%) |
| Intestinal obstruction | 1 (1.2%) |
| Cancer | 1 (1.2%) |
| Unknown cause | 49 (59.0%) |

**Supplemental Figure 1. Spline curve with Cox hazard regression between the MELD-XI score with 2-year all-cause mortality and major bleeding.**

**
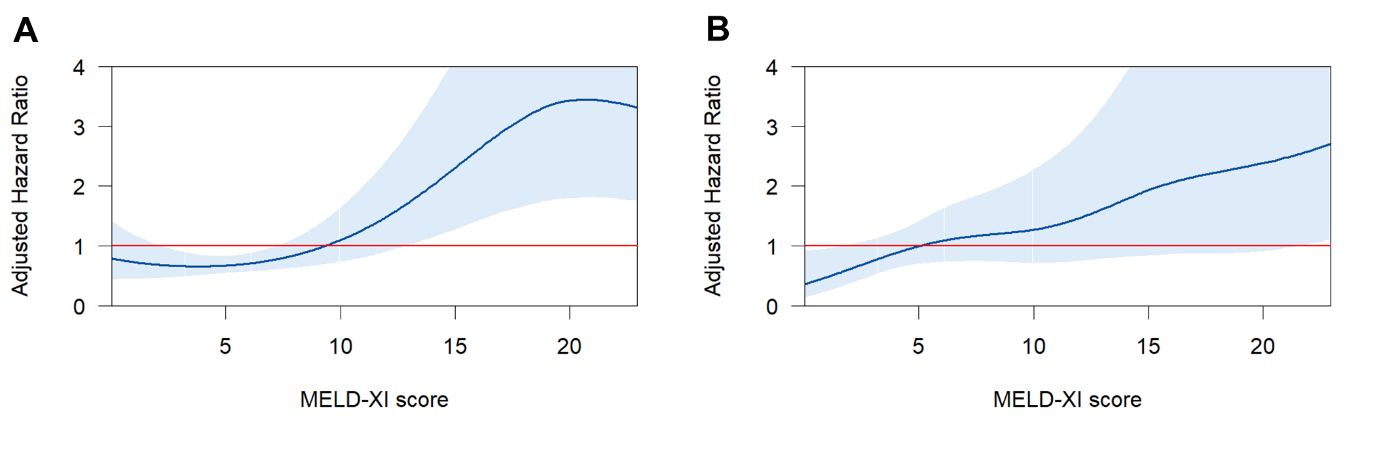
**

The non-linear relationship between the MELD-XI score and the all-cause mortality (A) and major bleeding (B) demonstrated a consistently increased adjusted hazard ratio for both outcomes as the MELD-XI score increased. Red horizontal lines represent the adjusted hazard ratio of 1.0. Blue lines indicate the estimated adjusted hazard ratio, and the areas shown in light blue represent a 95% confidence interval.

MELD-XI score, Model for End-stage Liver Disease eXcluding International normalized ratio score

**Supplemental Figure 2. Subgroup analysis of the secondary outcome in patients with a high MELD-XI score.**


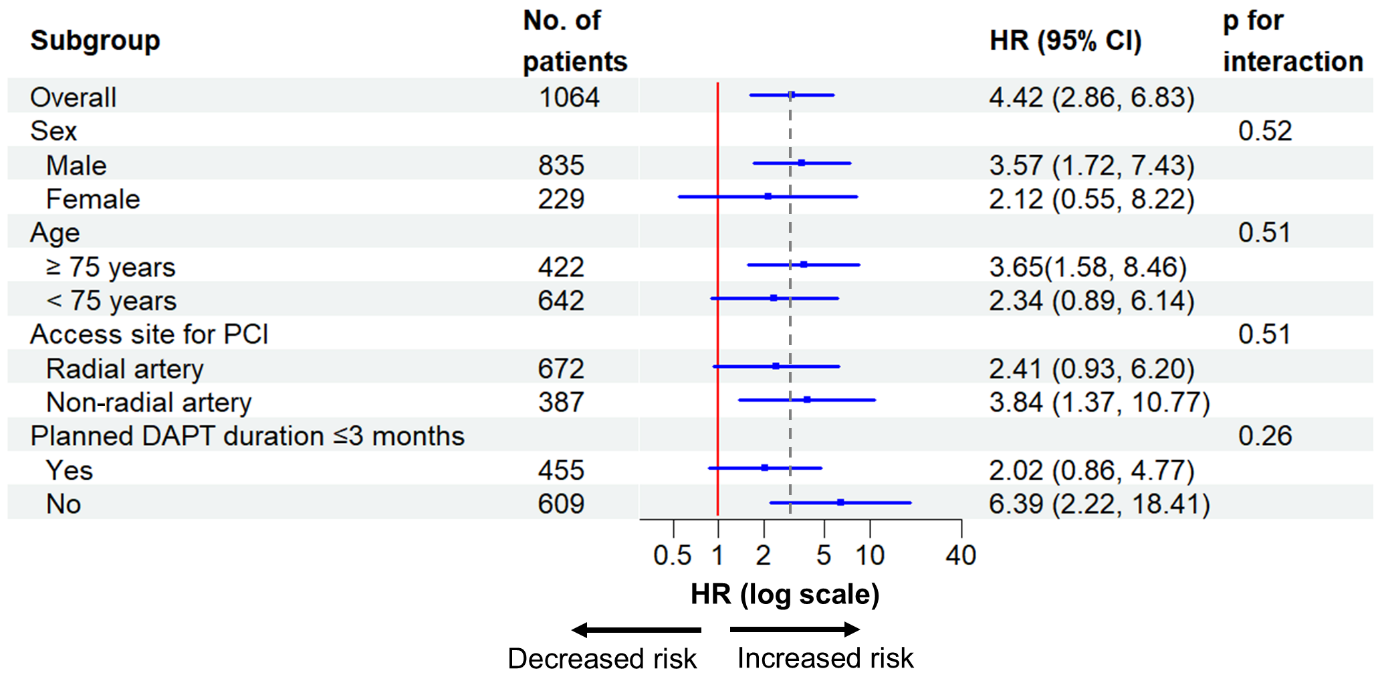


A forest plot illustrates hazard ratios for two-year major bleeding after PCI in patients with a high MELD-XI score. In each subgroup, hazard ratio and 95% confidence intervals are presented.

CI, confidence interval; DAPT, dual antiplatelet therapy; HR, hazard ratio; MELD-XI score, Model for End-stage Liver Disease eXcluding International normalized ratio score; PCI, percutaneous coronary intervention
